# Motor Alterations Induced by Chronic 4-Aminopyridine Infusion in the Spinal Cord *In vivo*: Role of Glutamate and GABA Receptors

**DOI:** 10.3389/fnins.2016.00200

**Published:** 2016-05-09

**Authors:** Rafael Lazo-Gómez, Ricardo Tapia

**Affiliations:** División de Neurociencias, Instituto de Fisiología Celular, Universidad Nacional Autónoma de MéxicoMéxico, México

**Keywords:** motor neuron degeneration, excitotoxicity, spinal cord, 4-aminopyridine, glutamate receptors, GABA_A_ receptor

## Abstract

Motor neuron (MN) degeneration is the pathological hallmark of MN diseases, a group of neurodegenerative disorders clinically manifested as muscle fasciculations and hyperreflexia, followed by paralysis, respiratory failure, and death. Ample evidence supports a role of glutamate-mediated excitotoxicity in motor death. In previous work we showed that stimulation of glutamate release from nerve endings by perfusion of the K^+^-channel blocker 4-aminopyridine (4-AP) in the rat hippocampus induces seizures and neurodegeneration, and that AMPA infusion in the spinal cord produces paralysis and MN death. On these bases, in this work we have tested the effect of the chronic infusion of 4-AP in the spinal cord, using implanted osmotic minipumps, on motor activity and on MN survival, and the mechanisms underlying this effect. 4-AP produced muscle fasciculations and motor deficits assessed in two motor tests, which start 2–3 h after the implant, which ameliorated spontaneously within 6–7 days, but no neurodegeneration. These effects were prevented by both AMPA and NMDA receptors blockers. The role of GABA_A_ receptors was also explored, and we found that chronic infusion of bicuculline induced moderate MN degeneration and enhanced the hyperexcitation produced by 4-AP. Unexpectedly, the GABA_A_R agonist muscimol also induced motor deficits and failed to prevent the MN death induced by AMPA. We conclude that motor alterations induced by chronic 4-AP infusion in the spinal cord *in vivo* is due to ionotropic glutamate receptor overactivation and that blockade of GABAergic neurotransmission induces MN death under chronic conditions. These results shed light on the role of glutamatergic and GABAergic neurotransmission in the regulation of MN excitability in the spinal cord.

## Introduction

Motor neuron (MN) degeneration in the spinal cord and/or in the cerebral cortex is the common pathological hallmark of MN diseases, which include a wide spectrum of heterogeneous neurodegenerative disorders. The loss of MNs leads to characteristic symptoms: initially hyperreflexia, muscle fasciculations, fatigue, weakness, cramps and later paralysis, and respiratory failure. Amyotrophic lateral sclerosis (ALS), spinal muscular atrophy, and spinal and bulbar muscular atrophy are some examples of MN disorders and, although having a relatively low prevalence, they all cause great morbidity and are uniformly fatal (Tiryaki and Horak, 2014). In spite of extensive research, no common cause of this selective MN degeneration has been identified and several hypotheses have been proposed, including glutamate-mediated excitotoxicity, mitochondrial dysfunction and energy failure, protein aggregation, endoplasmic reticulum stress, and RNA metabolism aberrations, among others (Dion et al., 2009).

Glutamate-mediated excitotoxicity is a key mechanism in neuronal death in most experimental models of MN disorders. It is possible to elicit excitotoxic neuronal death by different mechanisms, including: (1) pharmacological blockade of glutamate uptake; (2) stimulating endogenous glutamate release; and (3) overactivation of postsynaptic AMPA and NMDA types glutamate receptors (Mehta et al., 2013). Previous work from our group has established the differential efficacy of these strategies in triggering neurodegeneration in several regions of the rat nervous system *in vivo*. We have repeatedly shown that the infusion of the glutamate transport blocker pyrrolidine dicarboxylic acid by microdialysis or osmotic minipumps fails to provoke hyperexcitation and neuronal degeneration, even when inducing remarkable increases (up to 20-fold) in extracellular glutamate concentration, in the striatum (Massieu et al., 1995), hippocampus (Peña and Tapia, 1999), cerebral cortex (Tovar-y-Romo and Tapia, 2006), and spinal cord (Corona and Tapia, 2004; Tovar-y-Romo et al., 2009a). In the hippocampus, such increase due to transport blockade may even paradoxically protect against excitotoxicity due to inhibition of glutamate release by activation of presynaptic metabotropic glutamate receptors (Vera and Tapia, 2012).

Conversely, infusing 4-AP *in vivo* through microdialysis triggers behavioral and electroencephalographic seizures, as well as excitotoxic neuronal death, in the rat striatum (Morales-Villagran and Tapia, 1996) and hippocampus (Peña and Tapia, 1999, 2000). These effects are caused by a transient stimulation of the release of endogenous glutamate from nerve endings and the consequent overactivation of glutamate receptors, as demonstrated by the potent protective effect of the NMDA receptor antagonists MK-801 and CPP, and of the AMPA receptor antagonist NBQX.

4-AP perfusion by microdialysis in the lumbar spinal cord also induced a notable transient (30 min) increase in the extracellular concentration of glutamate as well as motor behavioral effects, such as fasciculations, twitching and muscle cramps in the hindlimbs, for up to 2 h, even when the rats were anesthetized, suggesting a muscular hyperexcitability state analogous to that of seizures; however, no significant MN degeneration was observed. In contrast, the microdialysis perfusion of AMPA induced MN death and hindlimb paralysis (Corona and Tapia, 2004), effects that were prevented by specific blockers of the Ca^2+^-permeable AMPA receptors and by intracellular Ca^2+^ chelators (Corona and Tapia, 2007). Since these excitotoxic effects induced by the microdialysis perfusion occur very rapidly, in less than 12 h, we developed a different model by chronically infusing drugs during several days through osmotic minipumps directly in the lumbar spinal cord tissue in behaving rats (Tovar-y-Romo et al., 2007). In this chronic experimental setting we showed that chronic AMPA receptor activation also induces MN degeneration and bilateral paralysis, while, as previously stated, glutamate uptake blockade does not cause MN degeneration even with a 12-fold increase in extracellular glutamate concentration (Tovar-y-Romo et al., 2009a).

In view of the above results, we considered relevant to elucidate if the chronic administration of 4-AP through osmotic minipumps in the spinal cord is also capable of eliciting MN degeneration and paralysis. We found that this chronic 4-AP infusion caused motor behavior deficits during several days and muscular signs of hyperexcitation, but since no MN degeneration or paralysis occurred we hypothesized that this might be due to a combined activation of both excitatory glutamatergic input on MNs and of GABAergic local inhibitory spinal circuits, whose function as modulators of motor activity has been clearly established (Martin and Chang, 2012; Ramirez-Jarquin et al., 2014). In fact, we and others have shown that the stimulatory action of 4-AP on the release of neurotransmitters is not specific for glutamate but involves also GABA (Jankowska et al., 1977; Morales-Villagran and Tapia, 1996; Peña and Tapia, 1999). Therefore, we also tested the effect of the GABA_A_R antagonist and agonist bicuculline and muscimol, respectively, on the excitotoxic effects of both 4-AP and AMPA. Our results indicate that chronic alterations of GABAergic neurotransmission are notably involved in the 4-AP-induced hyperexcitation mediated by overactivation of glutamate receptors and that may induce MN death.

## Materials and methods

### Animals

Adult Wistar male rats (280–300 g) were used in all of the experiments and were handled in accordance with the Rules for Research and Health Matters (Mexico) and with international standards of research animal welfare (including ARRIVE guidelines), and with approval of the Institutional Committee for the Care and Use of Laboratory Animals (protocol approval number RTI21-14). All animals were housed in a controlled laboratory environment with a 12 h light/dark cycle, and fed with regular animal chow and water *ad libitum*. All surgical procedures were performed under general anesthesia, and every effort was made to minimize animal suffering during experimental procedures.

### Drugs and osmotic minipump preparation

All drugs were dissolved in isotonic saline solution (SS). Osmotic minipumps (Alzet model 2004, volume ~250 μL, flow rate 6 μl/day) were filled with the different solutions containing the used drugs at the following concentrations: SS as control, AMPA 7.5 mM, 4-AP 35 mM, MK-801 1 mM, NBQX 1 mM, bicuculline methbromide 5 mM, muscimol 10 mM, or a mixture of drugs, as indicated in Results, 48 h before the surgical implantation, and were incubated at 37.0° in SS for flow rate stabilization. These concentrations were chosen on the basis of previous results from our laboratory (Peña and Tapia, 1999, 2000; Corona and Tapia, 2004; Tovar-y-Romo et al., 2007) and of preliminary experiments. AMPA, MK801, and NBQX were purchased from Tocris Bioscience, and 4-AP, bicuculline methbromide, and muscimol from Sigma Aldrich.

### Surgical osmotic minipump implantation

The procedure for osmotic minipump implantation was performed essentially as previously described (Tovar-y-Romo et al., 2007), with minor modifications. Briefly, animals were anesthetized with 5.0% isoflurane in a 95% O_2_/5% CO_2_ mixture and placed in a stereotaxic spinal unit. Later on, isofluorane concentration was gradually diminished to 1.5–2.0% as the surgery was performed. After shaving and disinfection, a median sagittal incision, 3.5–4 cm long, was made in the back of the animal and the underlying fascia and muscle tissue were dissected until appropriate visualization of the T10 vertebral lamina was achieved (at the spinal L3 level). The spinous process was removed with a drill, and a ~2 mm diameter hole was drilled in the right lamina until the spinal cord tissue was visualized and the meninges were carefully removed with a metallic hook. A stainless-steel screw (3.7 mm long, 1 mm diameter) was fixed in the left lamina. A fused silica glass filament probe (1 mm long, 50 μm internal diameter, 80 μm external diameter, VitroCom Inc.) was carefully advanced down into the spinal cord in a vertical fashion with the aid of the three-dimensional manipulator of the stereotaxic unit; previously, this glass probe was attached and glued to a plastic tubing ~1 cm long, and during surgery this tube was connected and fixed with cyanoacrylate glue to the osmotic minipump. During all these procedures great care was taken to avoid damage to the spinal cord tissue. Dental cement was poured and let dry on the T10 vertebra, to fix both the screw and the cannula on place. Osmotic minipumps were subcutaneously placed in the back of the animal, at the right side of the vertebral column. This procedure aims to directly and continuously administer the tested drugs in the spinal cord tissue, at a rate of 0.25 μl/h up to 25 days, in the vicinity of lamina IX, where motor neurons reside. Finally, the skin incision was closed with surgical stainless-steel clips, anesthesia was withdrawn, and animals received a single intraperitoneal antibiotic shot and were monitored until full consciousness was recovered.

### Behavioral assessment

Four to five days prior to surgery, rats were trained to walk during 120 s on an accelerating Rotarod (Columbus Instruments, USA), starting from 10 rpm (0.2 rpm/s of acceleration). Time to fall from the instrument was recorded, up to a limit of 120 s. Also, grip strength of both hind limbs was measured by placing the animals on their hind limbs on the metallic mesh of a grip strength meter (TSE Systems, USA) and gently pulling the tail to induce the animals to escape from the examiner; measurement was not considered if the animals, when attempting to escape, used their forelimbs. The maximum force displayed by the instrument in every trial was recorded in ponds, later converted to Newtons (1 pond = 0.009807 N), and normalized to values obtained the day before surgery (day 0). In both tests the best time, or the greater force, out of three trials was recorded. Great care was taken to avoid excessive distress in the animals, and appropriate time between trials was given to the animals to rest. These behavioral motor tasks were assessed on a daily basis for 5 or for 15 days (depending on the experiment) and, on these cutoff times, animals were sacrificed and fixed/perfused for histology.

### Histological processing

After the time for behavioral assessment was concluded, rats were perfused and fixed for histological analyses as previously described (Tovar-y-Romo et al., 2007). Briefly, animals were deeply anesthetized with an intraperitoneal injection of pentobarbital, the rib cage was cut to expose the heart, and a wide cut in the right atria was made. A needle, connected to a peristalsis pump, was inserted into the left ventricle and ~250 ml of ice cold normal saline were perfused, followed by ~250 ml of 4% paraformaldehyde in 0.1 M phosphate buffer. The back was dissected, the acrylic implant removed, and the lumbar spinal cord tissue was recovered by pushing it out of the vertebral canal with cold saline solution in a syringe. Tissue was postfixed for 48 h at 4.0°C, then dehydrated in increasingly concentrated sucrose solutions (10, 20, and 30%), and the region where the cannula was inserted was visually identified and used for study. Approximately 50 transverse sections (40 μm thick) were obtained in a cryostat, and the slices that showed the fused silica filament entry point were used for analyses and, of these, 15–20 slices were stained with cresyl violet (Nissl staining) for further analysis. The number of morphologically healthy MNs (multipolar neurons with clear cytoplasm, soma diameter >20 μm and distinguishable nucleus) was counted in the ipsilateral and contralateral ventral horns.

### Statistical analysis

All statistical analyses were carried out in GraphPad Prism 5 using a two way ANOVA (in the case of behavioral tasks) followed by a Bonferroni's *post-hoc* test, or a one way ANOVA (for number of healthy MN) followed by a Tukey's *post-hoc* test. A value of *p* < 0.05 was considered statistically significant.

## Results

### Chronic 4-AP infusion induces motor behavior alterations, that are enhanced by bicuculline

4-AP infusion caused motor behavior alterations in the hindlimbs of treated animals manifested as episodic muscle cramps and fasciculations that appeared frequently in the ipsilateral hindlimb. Animals seemed distressed when these alterations occurred, since they squealed, compulsively groomed the affected hindlimb, and even removed the overlying fur, and had occasional running fits. These episodic alterations started within 3 to 5 h after minipump implantation and lasted a few minutes with a variable frequency, but were often observed during or immediately after the rotarod tests. They progressively diminished in frequency, duration and intensity up to day 7 to 10, and subsequently were rarely observed.

**Figure 1:**
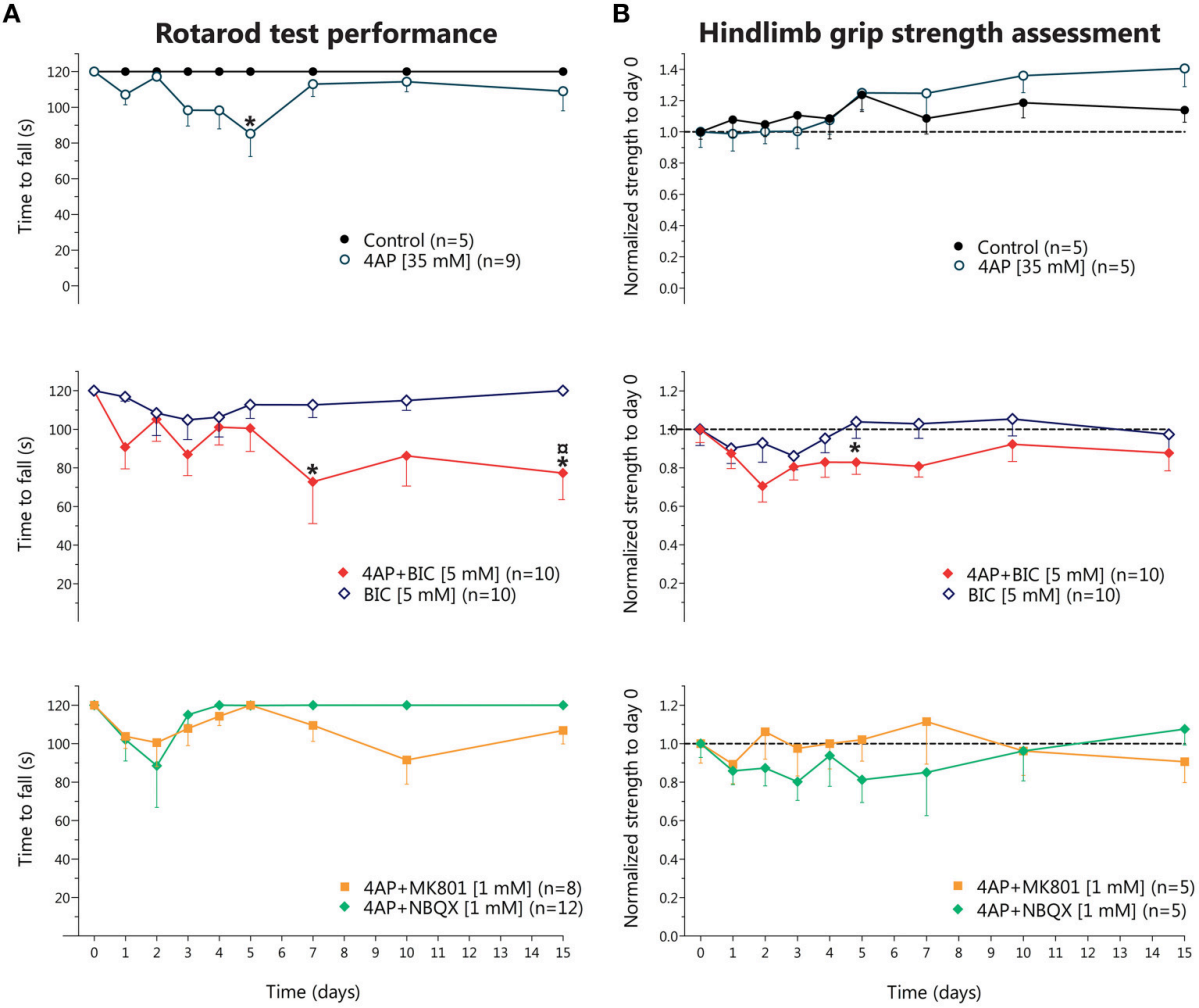
**Behavioral effects of chronic 4-aminopyridine infusion in the rodent spinal cord *in vivo***. **(A)** Results of rotarod test performance expressed as the time to fall (in seconds) in each of the experimental conditions. Top, 4-AP infusion causes a reversible decrease in the time to fall significantly different to Control only at day 5 (two-way ANOVA, Bonferroni's *post-hoc*; ^*^p < 0.05). Middle, bicuculline treatment did not result in significant changes, while 4-AP + bicuculline co-infusion produced an irreversible reduction in the time to fall, different to Control at days 5 and 15, and to 4-AP at day 15 (two-way ANOVA, Bonferroni's *post-hoc*; ^*^p < 0.05 vs. control; *p* < 0.05 vs. 4-AP). Bottom, Co-infusion of NBQX or of MK-801 with 4-AP partially improved time to fall, attaining results not different from Control or from 4-AP. **(B)** Results of hindlimb strength assessment normalized to baseline values (day 0, before osmotic minipump implantation). Top, Control group displayed a gradual increase in strength up to a ~10% at day 15 respect to baseline values, while 4-AP also displayed a non-significant gradual increase in strength up to ~30% at day 15. Middle, bicuculline treatment did not result in changes in strength in the 15 days of evaluation, while co-infusion of 4-AP + bicuculline resulted in a tendency to decrease strength during the 15 days of treatment, reaching significance only at day 5 (two-way ANOVA, Bonferroni's *post-hoc*; ^*^p < 0.05 vs. Control). Bottom, Co-infusion of NBQX or of MK-801 with 4-AP did not result in changes in strength.

These motor alterations were evidenced in the rotarod test as a progressive, but transitory, reduction in the time to fall, reaching significant difference only at day 5; in the grip strength assessment there were no changes, although we observed a slight non-significant increase in grip strength from day 10 until 15 (Figures 1A,B, top row). MK-801 co-infused with 4-AP almost completely prevented the fasciculations and muscle cramps induced by 4-AP, although the rotarod test and the hindlimb grip strength assessment did not show significant differences with respect to 4-AP alone (Figures 1A,B, bottom row). NBQX co-infused with 4-AP also partially suppressed the overexcitation induced by 4-AP and, again, the results obtained from the motor behavioral tasks were very similar to those of 4-AP treatment (Figure 1, bottom row). NBQX and M801 alone did not affect any of the parameters studied (not shown).

Then we tested the effect of bicuculline to explore the role of GABA_A_R in the 4-AP-induced motor alterations. Animals treated with bicuculline alone displayed only slight behavioral changes, such as occasional grooming and licking of the ipsilateral hindlimb, and did not alter significantly the rotarod performance nor the hindlimb grip strength. Co-infusion of bicuculline with 4-AP enhanced the intensity of fasciculations and the frequency of grooming induced by 4-AP; this was reflected in a decrease in the time to fall from the rotarod and in a trend to reduce the strength values as compared to 4-AP alone (Figure 1, middle row).

### Bicuculline, but not 4-AP, induces MN degeneration

**Figure 2:**
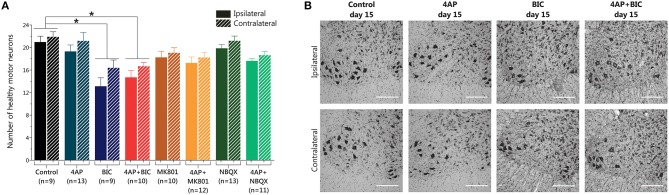
**Results of MN counting in the lumbar spinal cord after 15 days of 4-AP infusion and other compounds in the spinal cord *in vivo***. **(A)** Bicuculline-alone and 4-AP + bicuculline treatments induce a reduction in the number of MNs of similar magnitude (one-way ANOVA, Bonferroni's *post-hoc*; ^*^p < 0.001 vs. Control), while 4-AP infusion and the other shown treatments did not produce changes in the number of MNs. **(B)** Representative high-magnification microphotographs of Nissl-stained spinal ventral horns, where motor neurons reside (large, multipolar, intensely stained neuronal somas with prominent nuclei) after 15 days of the indicated treatments. Upper row show images of the ipsilateral side to the indicated infusion, and lower row show images of the contralateral side. (Scale bar, 250 μm).

In spite of the motor behavioral changes obtained with 4-AP infusion we did not find loss of MNs when we assessed the spinal cord tissue with Nissl staining (20.9 ± 3.1 vs. 19.3 ± 4.1 MNs ipsilateral side; 21.8 ± 2.7 vs. 21.1 ± 5.3 MNs contralateral side, compared to control). Neither NQBX nor MK-801, alone or in combination with 4-AP, altered the number of healthy MNs (Figures 2A,B). However, unexpectedly, bicuculline infusion caused a significant ~38% loss of MNs in the ipsilateral ventral horn with respect to control (13.1 ± 4.5 vs. 20.9 ± 3.1 MNs) and a ~25% reduction in the contralateral side (16.4 ± 4.1 vs. 21.8 ± 2.7 MNs); this reduction was also significant with respect to 4-AP alone, albeit only in the ipsilateral side. Co-infusion of 4-AP with bicuculline resulted in loss of MNs similar to that caused by bicuculline alone.

### Chronic GABA_A_R activation with muscimol does not prevent paralysis induced by AMPA receptor overactivation

**Figure 3:**
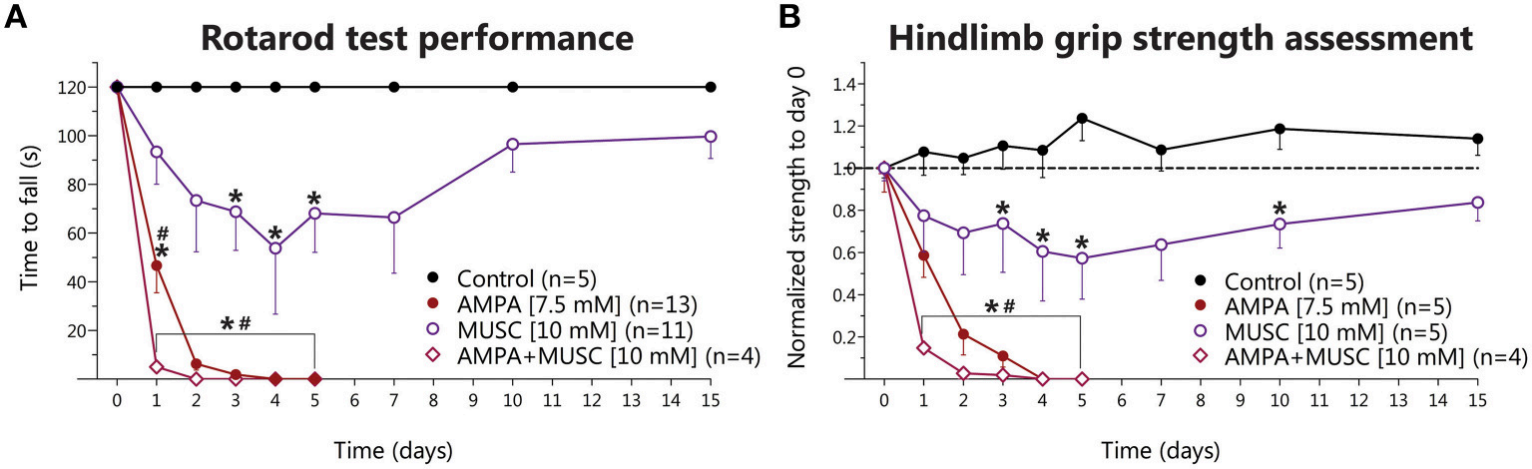
**Behavioral effects of chronic muscimol and AMPA infusion in the rodent spinal cord *in vivo***. **(A)** Results of rotarod test performance expressed as the time to fall in each of the experimental conditions. Muscimol causes a progressive reduction in the time to fall, which reverts late in the experimental timeframe of 15 days (two-way ANOVA, Bonferroni's *post-hoc*; ^*^p < 0.05 vs. Control), while AMPA infusion results in more severe and irreversible reduction in the time to fall in the 5 days of treatment. AMPA+muscimol coinfusion results in a further reduction in the time to fall, with an earlier onset, and also irreversible in nature (two-way ANOVA, Bonferroni's *post-hoc*; ^*^p < 0.05 vs. Control; ^#^*p* < 0.05 vs. muscimol). **(B)** Results of hindlimb strength assessment normalized to baseline values (day 0, before osmotic minipump implantation). Muscimol infusion also induces a reduction in hindlimb strength that reverts late in the experiment (two-way ANOVA, Bonferroni's *post-hoc*; ^*^p < 0.05 vs. Control), while AMPA and AMPA+muscimol infusions cause an irreversible, progressive and severe decline in the baseline strength (two-way ANOVA, Bonferroni's *post-hoc*; ^*^p < 0.05 vs. control; ^#^*p* < 0.05 vs. muscimol).

The above results indicate that 4-AP infusion produce a glutamate-receptor-mediated motor hyperexcitability state in the spinal cord *in vivo* albeit not sufficient to induce MN degeneration, whereas blockade of GABA_A_R was deleterious. So, to test the role of GABA-mediated inhibition in the excitotoxic effect of glutamate receptor overactivation, we infused muscimol alone or in combination with AMPA. Muscimol treatment provoked severe and long-lasting weakness in both hindlimbs, that in some animals manifested as paralysis with no other phenomena (such as autotomy, as is the case of AMPA-treated animals), starting at day 1 or 2, that progressively recovered throughout the 15 days' timeline but never reached control values again. Such motor alterations decreased the time to fall from rotarod significantly at days 3, 4, and 5 (Figure 3A), as well as a similar significant reduction in the grip strength at days 3, 4, 5, and 10 (Figure 3B).

As previously described (Tovar-y-Romo et al., 2007), chronic AMPA infusion induced a gradual and irreversible paralysis of the hindlimbs of all treated animals, manifested in the motor behavioral tasks as a progressive and irreversible diminution in the time to fall from rotarod and as reduction in the strength in both hindlimbs. The paralysis started on day 1 with the ipsilateral hindlimb and gradually progressed up to day 5, when animals were completely paralyzed in both hindlimbs. In this group, the experiments were stopped at day 5 because animals could no longer feed themselves and started to autotomize their hindlimbs. When muscimol was co-infused with AMPA, we observed an earlier onset and more severe paralysis, and an earlier, and greater reduction of the time to fall and in grip strength as compared to AMPA treatment, but no animals in this group displayed autotomy, as opposed to the AMPA-alone group. The values obtained were significantly different from control and from muscimol-alone treatment in both tasks (Figures 3A,B, respectively).

### Chronic GABA_A_R activation with muscimol does not prevent MN degeneration induced by AMPA

**Figure 4:**
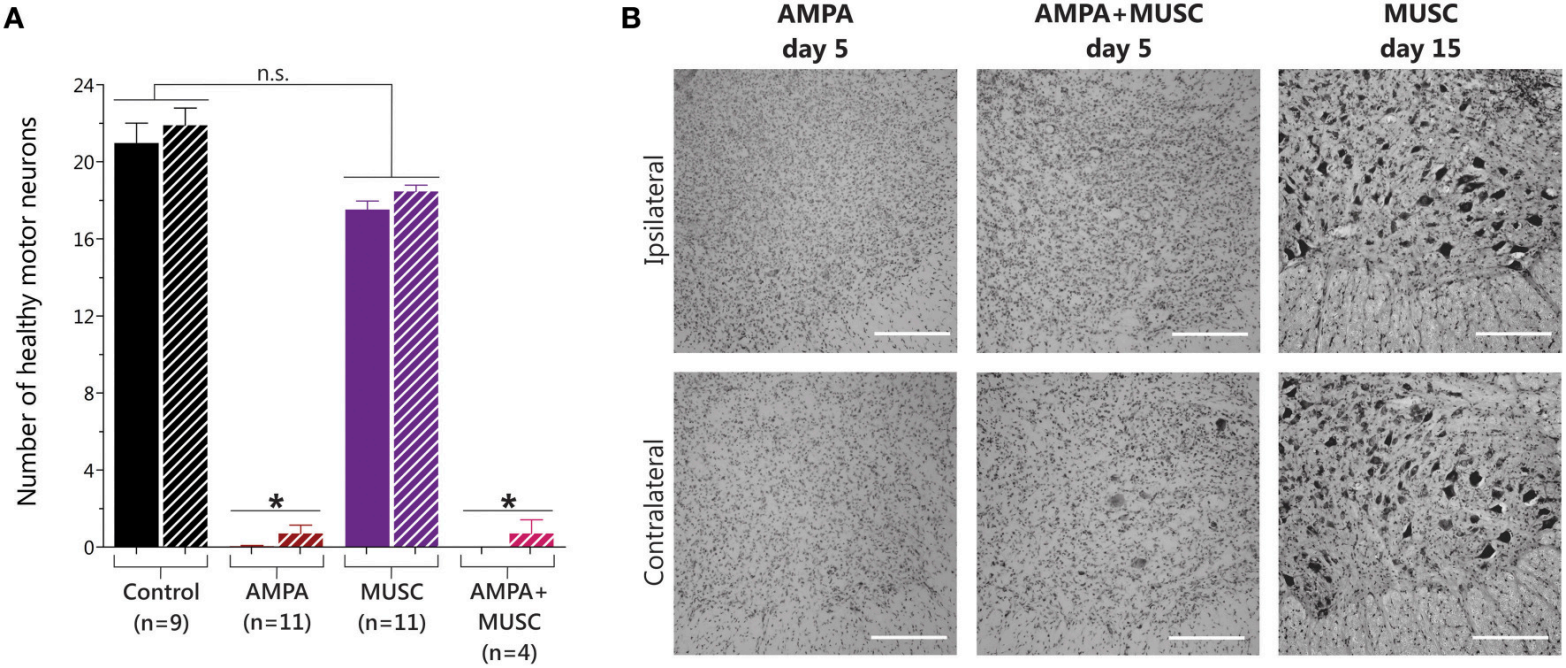
**Results of MN counting in the lumbar spinal cord after muscimol and AMPA infusions in the spinal cord *in vivo***. **(A)** 5 days of AMPA and AMPA + muscimol treatments caused a significant reduction in the number of healthy MNs, while 15 days of muscimol infusion did not change MN number (one-way ANOVA, Bonferroni's *post-hoc*; ^*^p < 0.001 vs. Control). **(B)** Representative high-magnification microphotographs of Nissl-stained spinal ventral horns after 15 days of muscimol infusion, or after 5 days of the indicated treatments. Upper row show images of the ipsilateral side to the indicated infusion, and lower row show images of the contrateral side. (Scale bar, 250 μm).

Histological analysis showed that muscimol infusion induced a non-significant slight reduction in MN number. In contrast, as expected, after AMPA infusion the paralysis observed correlated with the previously reported almost total loss of MNs (Tovar-y-Romo et al., 2007). Co-infusion with muscimol did not modify the remarkable MN degeneration induced by AMPA (Figure 4).

## Discussion

Excitotoxicity, a fundamental mechanism of MN degeneration, can be elicited by perturbing the presynaptic physiology in two means: (1) blocking the uptake of released glutamate, or (2) stimulating endogenous glutamate release. For the former, although there is evidence that implicate it as a mechanism of excitotoxic death in human MN disease (Rothstein et al., 1992), experimental studies using genetic knockout of the GLT1 glutamate transporter in mice have failed to causally link this finding to MN degeneration (Stoffel et al., 2004). Indeed, as was stated in the Introduction, work done by our group do not support glutamate uptake inhibition as an important mechanism in models of neurodegeneration *in vivo* in several regions of the rodent central nervous system in an acute (Massieu et al., 1995; Corona and Tapia, 2004; Tovar-y-Romo and Tapia, 2006) or in a chronic setting (Tovar-y-Romo et al., 2009b). In the present work we have used the later strategy in a chronic model of 4-AP administration in the spinal cord, in view that its acute perfusion caused behavioral evidence of hyperexcitability (muscle fasciculations and cramps), although animals were anesthetized, but no MN degeneration even when it transitorily stimulated up to 5-fold glutamate release immediately after its administration (Corona and Tapia, 2004). In addition, these results contrast with our previous findings showing that infusing 4-AP through microdialysis stimulates the release of endogenous glutamate and triggers immediate behavioral and EEG seizure activity and delayed excitotoxic glutamate-mediated neuronal death in the rat striatum (Morales-Villagran and Tapia, 1996) and hippocampus (Peña and Tapia, 1999, 2000). The effects of 4-AP on extracellular glutamate in the hippocampus were achieved almost immediately after its administration, and persistent activation (for at least 60 min) of the NMDA glutamate receptor is required for neuronal death (Ayala and Tapia, 2005).

Our present results show that, similarly to the acute administration, chronic 4-AP treatment *in vivo* failed to provoke spinal MN death and paralysis, although it did result in a hyperexcitability state manifested as muscle cramps and fasciculations. Though in this work we did not directly measure the frequency or intensity of those behavioral manifestations, these were enough to provoke changes in either behavioral motor tasks: in rotarod as a trend toward a decrease in the time to fall, and in hindlimb grip assessment as a trend toward an increase in strength, which reached a maximum at day 5. Although MN number was assessed after 15 days of continuous 4-AP infusion, and no changes observed, it is difficult to envisage that MN degeneration could occur at 5 days but not being detected at 15 days. In order to confirm that these results are secondary to an increase in glutamatergic neurotransmission, suggestively due to 4-AP-mediated stimulation of endogenous glutamate release, we examined the NMDA and AMPA types glutamate receptors and found that their chronic blockade prevented the motor behavior late in the experiment (until day 3–5). Therefore, our data indicate that glutamatergic neurotransmission in enhanced in the spinal cord by 4-AP, and that this may account for the behavioral hyperexcitability observed. The importance of glutamatergic transmission in the excitability control of spinal cord MNs is well established, both for the NMDA (Kudo and Yamada, 1987) and AMPA receptors (Pook et al., 1993), albeit these data were obtained through *in vitro* preparations.

Endogenous glutamate is not the only neurotransmitter which release is stimulated by 4-AP infusion, as GABA release has also been demonstrated by our group in the striatum (Morales-Villagran and Tapia, 1996), hippocampus (Peña and Tapia, 1999), and spinal cord (Corona and Tapia, 2004) *in vivo*. So, we hypothesized that endogenous GABA, and the consequent activation of its GABA_A_R, might counterbalance the potential neurotoxic effects of 4-AP-induced release of endogenous glutamate. The data we obtained from bicuculline treatment demonstrates the neurotoxic properties of chronic GABA_A_R blockade for the spinal cord MNs, though they neither develop as rapidly or as severe, both in motor behavior and in MN loss, as chronic AMPA infusion (Tovar-y-Romo et al., 2007). The behavioral data also reveal that GABA_A_R blockade potentiates the excitatory effect of 4-AP, but this is insufficient to enhance the bicuculline-induced MN degeneration. Thus, it seems that GABA_A_R activation due to 4-AP-released endogenous GABA has no role in the lack of neurotoxic action of chronic 4-AP treatment.

This finding was unanticipated, since GABA_A_R activation has consistently shown to be protective in models of neurodegenerative disorders where excitotoxicity has an important role (Rudolph and Knoflach, 2011). Also, GABA_A_Rs are expressed in spinal MNs (Bohlhalter et al., 1996), play an important role exerting tonic inhibition (Castro et al., 2011), and their activity has been implicated in MN disease (Ramirez-Jarquin et al., 2014). Indeed, several alterations in the GABA-mediated inhibitory control of synaptic excitability have been reported in models of MN disease: changes in subunit composition and ligand affinity (Carunchio et al., 2008) and decrease in GABA_A_R expression (Petri et al., 2003). These data suggest that inhibitory GABAergic neurotransmission failure may contribute to MN degeneration. Our results with bicuculline are in agreement with this statement, and are supported by a recent report in another *in vivo* model of MN excitotoxic death induced by a single intrathecal administration of NMDA and bicuculline (Ionov and Roslavtseva, 2012).

Since excitotoxicity implies an imbalance in excitatory and inhibitory inputs, with a resulting exaggeration of excitatory drive, we assumed that restoring inhibition by pharmacologically overactivating GABA_A_R in MNs might prevent the AMPA-mediated degeneration. We found that muscimol did not result in an amelioration of excitotoxic MN degeneration, although it prevented the muscle cramps and hindlimb autotomy induced by AMPA. In addition, we noticed an acceleration of paralysis progression as manifested by a steeper decline in the time to fall and in the hindlimb grip strength values. We suggest that the overwhelming inhibitory drive produced by muscimol induced a “functional paralysis,” without affecting the AMPA receptor activation causing MN degeneration. This conclusion is supported by the behavioral partial paralysis induced by treatment with muscimol alone. Although the duration of muscimol infusion was different from the AMPA + muscimol infusion, it is possible to compare both treatments because no MN death was observed after 15 days of muscimol alone, while MN loss was almost complete after only 5 days of AMPA + muscimol. Therefore, no further neuronal loss were probable after 5 days. To the best of our knowledge, this is the first report on the effects of GABA_A_R activation in the spinal cord *in vivo*, and indicates that inhibitory neurotransmission fails to prevent the excessive excitation caused by glutamate AMPA receptor overactivation. Another way to test the role of GABAergic neurotransmission in conditions of excessive excitatory drive would be to infuse 4-AP and muscimol, but muscimol would only add to the GABA_A_R activation already being achieved by the 4-AP-mediated release of endogenous GABA, and therefore the contribution of GABA_A_R activation could not be distinguished by this co-infusion. These results are largely in agreement with our previous work demonstrating a lack of protective effect of GABAergic neurotransmission-facilitating drugs in the NMDAR-dependent 4-AP-induced seizures and neurodegeneration in the hippocampus *in vivo* (Peña and Tapia, 2000), and with the lack of amelioration of clinical severity with the use of GABAergic drugs in ALS patients (Miller et al., 2001).

Although it might be possible that the stimulation of the release of other neurotransmitters may exert protective roles on spinal MNs *in vivo* (Ramirez-Jarquin et al., 2014) we did not explore that possibility. For instance, alterations in dopaminergic (Borasio et al., 1998) and serotonergic and noradrenergic (Bertel et al., 1991) neurotransmission have been documented in ALS patients, but there is scarce evidence of their role in excitotoxic MN degeneration. We propose that hyperexcitation produced only through direct receptor modulation (either inhibitory receptor blockade or excitatory receptor activation) may have a causal role in excitotoxic MN death, at least in relevant *in vivo* models. In view of our present, and of previous findings, it is challenging to establish a unifying model for the presynaptic mechanisms of excitotoxicity mediated by 4-AP throughout the mammalian nervous system. Better methodological strategies are necessary to dissect simultaneously the role of the many (if not every) neurotransmitter systems involved in the effects of 4-AP across the central nervous system.

In summary, we found that chronic 4-AP infusion in the spinal cord *in vivo* does not elicit MN degeneration but does produce motor behavior alterations, and these partially depend on ionotropic glutamate receptor activation, indicating that they are due to endogenous glutamate released by 4-AP. Also, we established that chronic GABA_A_R blockade produces MN degeneration and that this effect is not potentiated by 4-AP. However, chronic GABA_A_R activation produces transitory paralysis, probably because of overinhibition of MN activity, but no MN degeneration, and this effect does not counterbalance the excitotoxic effects of chronic AMPA receptor activation.

## Author contributions

RT designed and supervised the experiments, analyzed the results and wrote the final version of the manuscript. RL designed and carried out the experiments, analyzed the results and wrote the manuscript draft.

### Conflict of interest statement

The authors declare that the research was conducted in the absence of any commercial or financial relationships that could be construed as a potential conflict of interest.

## Abbreviations

NMDAR: N-methyl-D-aspartate glutamate receptor
AMPAR: α-amino-3-hydroxy-5-methyl-4-isoxazolepropionate glutamate receptor
GABA_A_R: γ-aminobutyric acid type A receptor
4-AP: 4-aminopyridine
MN: Motor neuron
ALS: Amyotrophic lateral sclerosis.
